# Respecting tribal voices in the development of a gestational diabetes risk reduction preconception counseling program for American Indian/Alaska Native adolescent females: a qualitative study

**DOI:** 10.1186/s12884-023-05850-9

**Published:** 2023-08-01

**Authors:** Kelly R. Moore, Sarah A. Stotz, Martha Ann Terry, Ellen W. Seely, Kelly Gonzales, Gale Marshall, Kristen J. Nadeau, Aletha Akers, Yesenia Garcia-Reyes, Denise Charron-Prochownik, For the Stopping GDM Study Group, Angela Brega, Laura Chalmers, Andrea Fischl, Heather Garrow, Jean Howe, Kristie McNealy, Nancy O’Banion, Jeff Powell, Susan Sereika, Howard Stein, Shelly Thorkelson, Xochitl Uribe-Rios

**Affiliations:** 1 Centers for American Indian and Alaska Native Health, University of Colorado Anschutz Medical Campus, Aurora, CO USA; 2 School of Public Health, University of Pittsburgh, Pittsburgh, PA USA; 3 Brigham and Women’s Hospital, Harvard Medical School, Boston, MA USA; 4 School of Public Health, Oregon Health & Science University - Portland State University, Portland, OR USA; 5 Two Feathers Media, LLC, Canton, NC USA; 6 Children’s Hospital Colorado, Anschutz Medical Campus, Aurora, CO USA; 7 Guttmacher Institute, New York, NY USA; 8 University of Pittsburgh School of Nursing, Pittsburgh, PA USA; 9 Department of Health Promotion and Development, Professor Nursing and School of Public Health, School of Nursing, University of Pittsburgh, 440 Victoria Bldg, Pittsburgh, USA

**Keywords:** Gestational diabetes, American Indians, Adolescents, Reproductive health

## Abstract

**Background:**

American Indians and Alaska Natives (AI/AN) are disproportionately affected by adolescent obesity, adolescent pregnancy and gestational diabetes mellitus (GDM). GDM is associated with increased risk for perinatal death, obesity, and subsequent type 2 diabetes (T2D) for the offspring. Moreover, mothers with GDM are also at increased risk for T2D post-partum. Yet few lifestyle interventions exist to reduce GDM risk prior to pregnancy. We describe the process of adapting an existing validated preconception counseling intervention for AI/AN adolescent girls at-risk for GDM and their mothers. Perspectives and recommendations were gathered from a diverse array of stakeholders to assure the new program called *Stopping GDM* was culturally responsive and developed with tribal voices and perspectives represented.

**Methods:**

We conducted focus groups and individual interviews with multiple AI/AN stakeholders (*n* = 55). Focus groups and interviews were digitally recorded, transcribed verbatim, and analyzed using a thematic content approach to construct cross-cutting themes across the focus groups and interviews.

**Results:**

Four key themes emerged reflecting issues important to planning a reproductive health intervention: 1) Limited awareness, knowledge, and health education resources about GDM; 2) The importance of acknowledging traditional AI/AN values and the diversity of traditions and culture among AI/AN tribes; 3) The need to cultivate healthy decision-making skills and empower girls to make safe and healthy choices; and 4) Lack of communication about reproductive health between AI/AN mothers and daughters and between AI/AN women and health care professionals.

**Conclusion:**

Findings have been used to inform the cultural tailoring and adaptation of an existing preconception counseling program, originally designed for non-AI/AN adolescent girls with diabetes, for AI/AN adolescents at-risk for GDM in future pregnancies.

## Background

The American Indian and Alaska Native (AI/AN) population is disproportionately affected by adolescent obesity, adolescent pregnancy, and gestational diabetes mellitus (GDM) [1–3]. American Indian and Alaska Native adolescents are twice as likely as adolescents from the general United States (US) population to be obese. Not unlike other North American adolescents, many AI/AN adolescents engage in early and unprotected sexual activity [4]. Yet adolescent pregnancy rates among AI/ANs are substantially higher than those of non-Hispanic white youth [5]. The increased occurrence of adolescent obesity is linked to higher rates of GDM among young AI/AN women, which in turn, are associated with increased risk for perinatal death, obesity, and diabetes in offspring [6]. The need for a preventive intervention to reduce GDM in AI/AN adolescents is compelling**.**

Significant gaps exist in the current body of knowledge about effective lifestyle interventions to reduce the risk of GDM. The majority of lifestyle interventions promote healthy eating and/or exercise during pregnancy among women at high risk for GDM [7–9]. A 2015 Cochrane review of 13 randomized controlled trials (RCTs) involving 4,983 women found no clear difference in the risk of developing GDM for women who participated in GDM risk-reduction interventions [10]. One explanation that has been proposed for the lack of clear benefit of these lifestyle interventions is that interventions initiated during pregnancy may occur too late to have any significant impact.

Few lifestyle interventions exist to reduce GDM risk prior to pregnancy. This gap exists despite some evidence that interventions administered prior to pregnancy can significantly reduce complications when pregnancy does occur. For example, for women with diabetes, receiving preconception counseling (PC) and delaying a pregnancy until it is medically safe and planned has been shown to reduce risk of complications [11]. However, a recently reported study of 228 women at high-risk for GDM in Finland randomized to a preconception lifestyle intervention or standard care, demonstrated no benefit in reducing GDM [12]. Moreover, although many of the lifestyle intervention studies conducted prior to or during pregnancy include diverse populations, none included sizable numbers of AI/AN individuals. Thus, the optimal lifestyle intervention strategies for AI/AN populations are unknown. The goal of this project was to address this gap.

We chose to adapt an existing, evidence-based preconception counseling program originally designed for non-AI/AN adolescents with diabetes to adapt and test, with the goal of reducing the risk for GDM among adolescent girls and to meet the needs of at-risk AI/AN adolescents and their adult female caregivers. Consistent with the position of the Association of Diabetes Care and Education Specialists (ADCES), formerly known as the American Association of Diabetes Educators [13], this study aimed to develop and evaluate a diabetes education program that could reduce health disparities experienced by a historically underserved group at high risk for GDM [13]. The position statement suggests cultural adaptation of diabetes education materials and programs is crucial to effectively meet the diabetes education needs of diverse audiences, especially those such as AI/ANs who have unique cultural values and often experience environmental challenges to diabetes prevention and management self-care practices [13]. These environmental challenges include lack of access to safe and sustainable water resources or water insecurity [14], environmental pollution, loss of traditional tribal lands and forced relocation that have ultimately resulted in the loss of AI/AN traditional healthy food practices such as hunting and fishing and access to AI/AN traditional healthy foods found in ancestral homelands [15, 16]. Moreover, food insecurity, extreme poverty and racism have also contributed to diabetes, obesity, and their complications among AI/ANs [17, 18].

In the development of interventions to decrease the risk for GDM and the tailoring of health promotion materials for population subgroups, it is important to consider the wide variety of factors that can contribute to success. The literature suggests that cultural adaptation specific to AI/AN audiences needs to consider traditional values: this is particularly important given the immense diversity of language and traditional practices within the AI/AN population. Moreover, the process of adapting educational materials needs to go beyond the inclusion of culturally-appropriate images and graphics [19, 20]. When developing reproductive health interventions for AI/AN women, consideration must also be given to the legacy of past medical programs designed to limit the reproductive choices of AI/AN women, including sterilization without consent [21].

Research into these issues has documented that AI/AN women may disengage with healthcare including reproductive health care. This lack of engagement is often due to their perceived experiences of racial discrimination and concern about being stereotyped and stigmatized. This may result from explicit or implicit provider bias and systemic inequities within healthcare [22]. However, several studies have shown that, among diverse groups, culturally responsive healthcare programming is a promising approach to mitigate the impact of healthcare inequities and may help to improve patient engagement, adherence, and outcomes [23]. Given the sensitive nature of reproductive health and the lack of culturally responsive programming available to AI/AN women, there is an urgent need to understand and contextualize the lived experiences and perceptions regarding reproductive healthcare among this population and specifically with regard to reducing risk of GDM.

We elicited advice and feedback from multiple AI/AN stakeholder groups to inform the development of a GDM risk reduction intervention designed to educate nonpregnant adolescent women at increased risk for GDM. We sought to adapt an existing, evidence-based program entitled *READY-Girls* (Reproductive-health Education and Awareness of Diabetes in Youth for Girls) [24]. *READY-Girls* is a validated, theory-based, PC program for adolescents with diabetes. The booklet-based educational curriculum aims to raise awareness about diabetes and pregnancy, prevent unplanned pregnancies, and reduce pregnancy complications among non-AI/AN adolescents with diabetes. *READY-Girls* is conceptually guided by the Expanded Health Belief Model (EHBM), which posits that adoption of target behavioral outcomes is predicted by health-related beliefs, self-efficacy and behavioral intentions [25, 26]. Three randomized controlled trials conducted with adolescent women ages 13 to 19 years found that the program increased knowledge, attitudes, contraceptive use, and initiation of preconception health discussions with health professionals [27–29].

This manuscript represents a component of a larger 5-year study that included an initial formative research phase to develop/adapt the *Stopping GDM* program, a second phase for a pilot and randomized controlled trial, and a final phase for program dissemination. The purpose of this study was to explore cross-cutting thematic perspectives from a diverse array of stakeholders on their awareness and understanding of GDM and reproductive health and how to culturally adapt *READY-Girls* – an existing validated PC program for non-AI/AN adolescents with diabetes for AI/AN girls at-risk for GDM and their mothers. Perspectives and recommendations were gathered from a diverse array of stakeholders as it was critical to assure the program was culturally responsive and developed with AI/AN community voices and perspectives represented, as suggested by the literature [30]. The stakeholders included elected tribal leaders; AI/AN health professionals; and, experts in AI/AN health care delivery, pediatric and gestational diabetes, adolescent health, and reproductive health. Additionally, we conducted focus groups and interviews with other key stakeholders: AI/AN mothers of adolescent girls, AI/AN women with a personal history of GDM or current diagnosis of type 2 diabetes (T2D), and AI/AN girls who were at risk for GDM. Findings from the focus groups and interviews held with each stakeholder group are reported elsewhere [31–34]. Each focus group informed the cultural adaptation of *READY-Girls* for AI/AN adolescent girls at-risk for GDM using the unique perspectives and recommendations of each distinct stakeholder group. Only overlapping themes across the diverse stakeholder groups are highlighted in this paper. The process for adapting the *READY-Girls* intervention for AI/AN adolescent girls at-risk for GDM and their mothers is also described.

## Methods

### Study design

The constructivist case study methodology used in this adaptation project allows health education researchers to study complex phenomena within their contexts and develop interventions and education programs [35]. The single instrumental case study design was chosen to provide a rich description to help understand the multifaceted issues of key stakeholder perspectives of a gestational diabetes risk reduction education program for AI/AN girls [36]. The single case is defined as the program itself, entitled *Stopping GDM* [37]. Focus group and individual interviews are well suited as a method for a constructivist case study project in order to help the researchers generate ideas and develop interventions [38] and interviews are usually the most important type of data collected in case study research [37].

### Data collection

Semi-structured focus group and individual interviews allowed the interviewer to refer to a prepared moderator guide of open-ended questions, with additional follow-up questions and probes which varied depending on the response of the interviewee(s) [38]. Examples of moderator guide questions for each of the groups can be found in Table 1. In addition, during the focus groups, video snippets and booklet excerpts were used to elicit conversation and impressions of the original *READY-Girls* program. Focus groups were all conducted in person and digitally recorded. A note taker assisted the moderator. Several of the individual key informant interviews were completed via web-based teleconference. Focus groups and interviews were digitally recorded and transcribed verbatim by a professional transcription service. No identifying information was collected during the focus groups and interviews; where present, identifying information, including names, was excluded from the transcripts. All digital recordings were destroyed following the completion of transcription. Some individual key informants and some focus group participants also completed markups of selected pages from the *READY-Girls* book to provide additional feedback. These study participants were instructed to not include their names on the markups.

**Table 1:** Example moderator guide questions for each group of interviewees

Participants	Moderator Guide Questions
Tribal and urban Indian health administrators and elected tribal leaders	1. Are you familiar with gestational diabetes or GDM? 2. Are there effective/successful programs in your community that address sexual health/sex education with AI/AN teen girls? Mothers? If so, what makes them effective/successful? 3. Are there effective/successful programs in your community that address diabetes prevention? 4. What do you know about teens in your community that would help us to modify this intervention to help them understand the importance of managing healthy lifestyles to prevent GDM? 5. What do you know about your tribal culture that would help us as we modify this intervention to make it more culturally relevant? How should it be included? 6. Tell me about any “coming-of-age” rituals which are practiced in your community
Expert panel of health care professionals	1. Please tell me your name and how you interact with AI/AN teens 2. How comfortable do you think the teen girls you know will be getting this kind of information? From whom? Will they be comfortable discussing it with those folks? With their friends? Their family? 3. What other sexual health and sexuality education programs do you know about that are successful with and respected by AI/AN girls and their moms? 4. What else can you tell us that you think will help us develop these materials into something that AI/AN teen girls will like, look at, talk about?
AI/AN girls (12–20 years of age) at risk for GDM and AI/AN adult female caregivers	1. How can a woman stay healthy during her pregnancy? 2. How does weight effect a pregnancy? 3. What do you know about gestational diabetes? 4. What do you know about preconception counseling? 5. What do you know about your tribal culture that would help us as we modify this intervention to make it more culturally appropriate for/acceptable to you and other girls like you? How should it be included? 6. If you had questions about gestational diabetes, pregnancy, puberty, birth control, who in your community would you ask? Where would you go to get this information?
AI/AN women with history of GDM	1. How can a woman stay healthy during her pregnancy? 2. How does weight affect a pregnancy? 3. What did you know about gestational diabetes before you had it? 4. What would you have liked to have known about gestational diabetes? 5. Where did you find information about GDM? What is helpful/useful? 6. What was good about the information you had and what wasn’t? 7. Do you think women with gestational diabetes are adequately educated/supported? If so, what is available to them? If not, what would be helpful to be available to them? 8. What do you know about preconception counseling? 9. What do you know about your tribal culture that would help us as we modify this intervention to make it more culturally appropriate for/acceptable to young women/teenagers in your community? How should it be included? 10. If a woman in your community had questions about gestational diabetes, pregnancy, puberty, birth control, where could she go and who could she ask? How could she get this information? 11. Do you feel that gestational diabetes can be prevented? If so, tell me about how you think it could be prevented

### Sample/setting

Stakeholders are outlined in Table 2. Both lay and expert stakeholders were included in this data set. Lay participants were composed of AI/AN women with a history of GDM or a current diagnosis of T2D and AI/AN mother/daughter dyads. These participants were recruited using maximum variation sampling [39] via word-of-mouth and posted flyers. Lay participants were interviewed in a focus group format when logistically possible, although some individual interviews were conducted because of scheduling and location issues. The lay focus groups were conducted in urban AI/AN community centers in Denver, CO, and Portland, OR. These centers provide social services such as job placement, community food banks, and clothing and serve as a cultural gathering place for urban AI/AN people and families from multiple tribal affiliations. Girls were required to be 12 – 18 years of age; AI/AN; accompanied by an AI/AN adult female caregiver (e.g., mother, grandmother, aunt or older sister); overweight with a BMI ≥ 85^th^ percentile for age from self-reported weight and height (obtained by the study coordinator in telephone screening for eligibility); fluent in English; and, not have type 1 or type 2 diabetes. Women were ≥ 18 years of age; AI/AN; fluent in English; and, had a history of GDM or a current diagnosis of T2D. Expert participants included both tribal leaders and health care professionals. Elected tribal leaders and AI/AN health care system administrators were recruited at the 2015 National Indian Health Board conference through flyers in registration packets and word-of-mouth on-site. The tribal leaders focus group was conducted in a meeting room at the conference hotel in Washington, DC. The focus group participants were 18 years of age or older; AI/AN; and an attendee at the National Indian Health Board conference. An expert panel of health care professionals was identified by purposive sampling [39] in order to obtain an intentional sample characterized as health care professionals with expertise in serving AI/AN communities. An obstetrician, registered dietitian, midwives, nurses, pediatricians, and endocrinologists (both pediatric and adult) were included. Some also had adolescent health and mother-daughter communication expertise. Seven of the 16 health care professionals were themselves AI/AN. An in-person focus group was convened in Aurora, CO, on the University of Colorado Anschutz Medical Campus. However, several of the expert participants were interviewed via web-based conference call technology using Zoom for logistical reasons.

**Table 2:** Focus group and individual interview stakeholders

Stakeholders	Data Collection Method	n
AI/AN girls (12–20 years of age) at risk for GDM	Focus groups	13
AI/AN mothers or other adult female primary caregivers (e.g., grandmothers, aunts or sisters)	Focus groups	9
AI/AN women with history of GDM	Focus groups and individual interviews	5
AI/AN tribal and urban Indian health administrators and elected tribal leaders	Focus group	12
Health care professionals/experts in AI/AN adolescent health, pediatric diabetes, reproductive health, GDM, mother/daughter communication (44% of these individuals were AI/AN)	Focus groups and individual interviews	16

### Analysis

Thematic analysis was used to produce findings from both the focus group discussions and the interviews. This approach uses the questions as a guide to identify themes within and across transcripts, to create codes and to assign codes, both to themes related to the core questions and to additional themes that emerged from the data.

Initial analysis, conducted by MAT, involved reading one focus group transcript in order to develop a codebook and apply the resulting codes. The same transcript (uncoded) was shared with SS, who used the working version of the codebook to apply codes, after which MAT and SS compared the transcript for concordance. Differences were discussed and consensus was reached, by agreeing, revising the code’s definition or creating a new code. A combination of inductive and deductive coding approaches was employed. The deductive coding approach was guided by interview guide themes and a codebook was generated for use across all transcriptions. The thematic coding approach included coding data, categorizing the codes, and reorganization of the categories into thematic representation through a series of assertions and interpretations [38, 40]. Using this method, data were compared across transcriptions to find similarities and differences, recognizing too, the researcher’s own observations, ideas, and intuitions influenced this process [40]. The coding strategy did not include line-by-line coding as this level of abstraction did not best represent the data. Rather, codes were applied to fully developed thoughts/sentences/ideas about participants’ shared expectations and experiences [40]. Initial coding involved both deductive and inductive coding, in that codes were derived from discussion guide questions and created from emerging themes as transcripts were read. A second iteration of coding allowed researchers to identify redundant codes and to posit relationships between codes. The process of consensus coding was used to establish inter-rater reliability. That is, after independently coding the same transcripts, MAT and SS met to compare where codes had been applied in each of the transcripts. Where differences were identified, MAT and SS discussed the content of the data and determined whether to 1) use the code applied by MAT, 2) use the code applied by SS, 3) add a new code; or 4) revise the definition of the initial code.

### Ethical oversight

This study was approved by the Colorado Multiple Institutional Review Board (COMIRB). A waiver of documentation of informed consent was granted. A COMIRB-approved information sheet was distributed to all study participants at the beginning of each focus group/interview session. All participants were given time to read the information sheet and ask questions prior to their session.

## Results

### Thematic overview

Four key themes emerged reflecting issues important to planning a reproductive health intervention: 1) Limited awareness, knowledge, and health education resources about GDM; 2) The importance of acknowledging traditional AI/AN values and the diversity of traditions and culture among AI/AN tribes; 3) The need to cultivate healthy decision-making skills and empower girls to make safe and healthy choices; and 4) Lack of communication about reproductive health between AI/AN mothers and daughters and between AI/AN women and health care professionals.

### Limited awareness, knowledge, and resources about GDM

All stakeholders expressed the belief that awareness and knowledge about GDM is limited within the AI/AN community. The lack of knowledge was thought to be at least partially attributable to the lack of education about GDM offered *prior to pregnancy*. Stakeholders cited a lack of education provided by schools, community programs, and within health care settings.

Many women with a prior history of GDM shared they had never heard of GDM until they were diagnosed with it. This was true even among women with a strong family history of diabetes, as shared by one woman who stated:

*“I had no idea what it was. I knew my grandma was diabetic and my mom was diabetic, but that was all I knew. I didn’t know that you could be diabetic during pregnancy, or that it could come on with a pregnancy. I had no idea.”*

Although knowledge levels among AI/AN girls varied, in general, adolescent stakeholders were more knowledgeable about GDM than the adult women, including adult women with a history of GDM. Some girls were highly knowledgeable about GDM: others had only a vague knowledge of “pregnancy diabetes”. Most importantly, few had little or no knowledge about GDM risk factors, self-care recommendations, or the short-term or long-term implications of GDM for moms and their babies.

Stakeholders identified a number of existing barriers to increasing knowledge about GDM prevention and management. Health care professionals shared that there were limited health education resources about GDM, as opposed to resources about diabetes in general, and few resources that have been adapted specifically for AI/AN populations.

*“…we try to give* [women] *a handout on how to prepare for taking care of their diabetes prior to getting pregnant. It’s more targeted for people who already have diabetes… not for people who are at risk of GDM”.*

Comments from women who previously had GDM complemented those of health care providers. Women lamented that despite the increased risk of GDM among AI/AN populations, information about GDM risk and prevention through lifestyle modification was not accessible prior to diagnosis. Women believed educating AI/AN girls about how to reduce their risk was of particular importance.

*“I wish I would have known the effect of my diet more. That was just never talked about. And nobody really exercised when I was younger. We exercised at school in gym class, and that was it.”*

AI/AN girls and their adult female caregiver’s limited awareness and knowledge of GDM is at least partially attributable to the lack of educational materials and programs related to this topic that are offered *prior to pregnancy*.

### The importance of acknowledging traditional AI/AN values and the diversity of traditions and culture among AI/AN tribes

Stakeholders spoke to the incorporation of traditional AI/AN values, the concept of balance and honoring the traditional roles of women and family.

Stakeholders shared the importance of “balance” regarding the mind, body and spirit and that these aspects of health should be considered when developing education programs adapted for AI/AN youth and other respective AI/AN audiences. The youth, tribal leaders, mothers, and health professionals spoke also about the importance of family and community for learning and maintaining healthy behaviors for a balanced life. One health professional shared:

*"This program can be tailored for our community to be more holistic and* [to include]* all parts of your life...the physical, emotional, and spiritual."*

One tribal elder described the importance of family.

*“A larger picture about healthy relationships within American Indian community, and for me…is always about showing, about telling that cultural piece about who you are - like for me as an Anishnaabe, the role of men and women and that balance, and that is what I would want to promote as a tribal elder/grandma/mother for my daughter.”*

Further, regarding balance, stakeholders shared that GDM risk reduction should focus on healthy eating, being more physically active, and healthy weight management rather than weight loss and the deficit and shame-based approach generally used within the biomedical field that focuses on achieving weight loss. The women described that healthy eating means learning to harvest and properly prepare “traditional foods”. With regard to physical activity, the women cited that a strengths-based culturally relevant approach emphasizes activities that connect individuals to traditional cultural practices such as gardening, regalia making, planting and harvesting herbal and plant remedies, dancing, and traditional AI/AN games. These ideas were echoed by tribal leaders, mothers, and non-AI/AN health care professionals with experience serving AI/ANs using culturally responsive approaches. Health care professionals also shared the benefits they have seen in educational materials that promote adult female caregivers to uniquely cultivate and instill cultural values in their daughters that help them to grow into healthy women and community members.

*“I mean, I’m hoping that I won’t have it* [GDM] *again, because I also think that part of having your spirituality and stuff and being balanced* [is] *that this* [is] *a journey. You design your journey and maybe this is just a warning in my life, having gestational diabetes. That I should wake up, and the foods, all the processed foods, and things like that are not good for us. And we should get the word out, you know?”* (woman with history of GDM)

*“…a fundamental part of teaching communication skills to parental caregivers…is making it clear to them that what we don’t teach* [parental caregivers’] *values. that’s not our job,* [as health care providers] *and that they* [parental caregivers] *need to understand their values in order to have these conversations with their kids. We’re not going to tell people what their values are, but I do think as part of this, we need to make sure that we make it clear that understanding one’s own values and what that brings to conversations is important.”* (health care professional)

Additionally, women shared the importance of teaching concepts of women’s health, reproductive health, and “creation” in terms of AI/AN values.

*“And I think kids will listen to that. They’ll listen to that, if you mix it up – because you asked about culture. I think that if you mix it up with a creation story somehow – because we listen to stories … And our kids, they listen to what we say, so we teach them with words. And I just think that if you were able to share with them some kind of creation story, and just intermingle it with the information about how their eggs are already there and need to stay healthy, then that’s part of creation. They’re making their own creation story. I think that you might ring some bells.”* (woman with history of GDM)

### The need to cultivate healthy decision-making skills and empower girls to make safe and healthy choices

Tribal leaders, AI/AN girls, and health care professionals emphasized the importance of empowering girls to make healthy choices related to their reproductive health decisions and healthful lifestyle choices that influence GDM risk. Commonly cited reproductive health decisions included decisions about romantic and sexual partner selection as well as engagement in sexual behaviors that increase unintended pregnancy risk. Commonly cited lifestyle choices included decisions about diet and physical activity.

Tribal leaders and healthcare professionals shared the belief that nurturing a sense of competence to make healthy choices coupled with knowledge were critical. As one girl at risk for GDM said,

*“I think if you have good self-esteem…if you have a very solid foundation of who you are and how to deal with things,* [you will make better choices]*.”*

Another girl at risk for GDM emphasized the critical value of education and skills-building through school-based programs,

*“…like a sex ed and a health class, and in the health class, they* [should]* teach good coping skills, like self-empowerment.”*

In addition to schools, these stakeholders envisioned that entire families and communities should be included in education programming as a means to ensure broader support for girls in making healthy choices.

*“I think mother-daughter communication is good, but also the whole family – like it takes a whole village to raise a family or child – and the community wants to help. That is the way we are.”* (mother of girl at risk for GDM)

Stakeholders shared a myriad of barriers that may stymie girls from making healthy reproductive choices including lack of access to information on safe-sex, lack of access to reproductive health services, and personal experience with intimate partner violence. The stakeholders also described that some AI/AN youth may be subjected to abuse that may further undermine healthy reproductive choices by disempowerment and erosion of healthy self-esteem. Indeed, the cycle of trauma is described among AI/ANs and cited as a prominent determinant of AI/AN health.[41, 42].

*“I know there’s* [more] *Native American* [AI/AN] *women who are raped and sexually molested and abused. When* [the book] *said, “In becoming a woman, you will have many choices,” I just think it sounds better if you say, “You will make many choices.” Because it gives them some power back and it lets them know, “You will make these choices.” If a girl is sitting there and she’s already insecure and she’s already been abused, and she already doesn’t know her body and she’s scared to even talk about sex and stuff like that, or whatever, she’s probably not going to want to hear, “You will have many choices.”* (health care professional)

Additional challenges include access to healthy food, safe places to engage in physical activity, and loss of traditional foods and food acquisition practices. One girl at risk for GDM shared a story about her older sister who was diagnosed with obesity:

“*Yeah, I don’t know. I think that would be good to have this information brought to the nutritionist and stuff. Because she* [my sister] *was seeing – because she [was] diagnosed with being obese, so we went to see a nutritionist… I know we went a few times, but then she got tired of hearing the same thing from her, because it was more of the nagging, “Are you doing this? Are you doing that?” And there’s just a lot of things to play into that role and it all goes back to eating healthy. And a lot of Natives* [AI/AN] *can’t because it’s expensive to try and eat healthy. She* [the nutritionist] *offered us food banks and things like that, which is fine, but when you go to the food banks, they don’t offer you healthy food and stuff like that”.*

### Lack of communication about reproductive health between mothers and daughters and between AI/AN women and health care providers

All stakeholders cited lack of open communication about reproductive health within families, among community members, and between health care professionals and patients. This significantly contributes to the lack of knowledge about GDM and the lifestyle factors that could reduce the risk of diagnosis.

Some AI/AN adult participants shared they had excellent support from their health care provider after they were diagnosed with GDM. However, prior to their diagnosis of GDM, and for those who have never experienced GDM, AI/AN adult females reported there was a lack of discussion about reproductive health and GDM with their health care provider.

*“I remember doing that test. I just thought – the way my doctor said, “Oh, this is just the regular routine diabetes test we do with all pregnant women.” But didn’t use the term “gestational.” My original doctor didn’t explain anything to me. So I was just totally oblivious to it. And I was like, oh, just another test. Didn’t really pay no mind or worry about it, or think that I had to worry about it, or prepare for it, or anything. I was just thinking, “No, I’m not diabetic. I know this.” I know it runs in my family, and almost the majority of all my family members have it on my mom’s side. I just didn’t know anything. I just thought it was just diabetes. For sure no one told me or talked to me about this before* [GDM diagnosis].”

Lay stakeholders shared that conversations about reproductive health and sex can be “embarrassing” and many parents and teenagers are “shy” to discuss this topic. Lay participants with children shared that they strive to have more open conversations with their children (both sons and daughters) on practicing safe sex, having their own healthy intimate relationships, and serving as a supportive resource for their children. They suggested that as children themselves, they did not have this open communication about sex or reproductive health with their mothers, which was often the motivation of their own desire to communicate openly with their children on these topics.

*“There’s more* [communication] *now than there was when I was younger. Nobody wanted to talk about it. They didn’t talk to us about anything when we were young. Me and my cousins - we didn’t know much about it. What I learned, I learned at school or from friends. And I went to school with a lot of other Native* [AI/AN] *girls, but they were kind of in the same boat as me. We kind of all winged it. And now? Oh, I think it’s completely different. I talked to them* [my children]. *I’m really open with them. They’re open with me. My daughter was 12 – my youngest daughter – was 12 when she started menstruating. And she came to me, and we went over tampons, pads, and she’s tried both.”*

Health care provider stakeholders shared they routinely and easily have discussions about reproductive health with their adolescent female patients. They also make an effort to ensure parents and families are comfortable, confident, and have the resources they need to engage in these difficult conversations.

*“I promote open communication – since a family that’s engaged is going to improve healthy lifestyles overall, so that is what my take-home message needs to be, always…… As* [for] *the information we’re providing, we’re helping and supporting the girls. We’re helping getting tools to the mothers. But it’s important as part of teaching these skills, they understand that each of them are unique individuals who are doing their best by one another, but they may ultimately have different approaches. We’re not going to tell people what their values are.”*

## Discussion

### Process for incorporating stakeholder voices into *Stopping GDM*

The input and feedback from the focus groups and interviews informed the modifications to the *READY-Girls* program and the adaptation for the new online program *Stopping GDM* consisting of a video and eBooklet. The video and eBooklet contain parallel information on GDM, its risk factors, and recommendations for a healthy lifestyle to reduce the risk for GDM. The information was also tailored for an AI/AN audience to increase its cultural relevancy. We used what some researchers term ‘surface structure’ modifications that retain the core elements of the original evidence-based intervention (in this case, *READY-Girls*) while adding culturally relevant elements [30], such as examples of traditional foods in the Native Plate in the section of the eBooklet on healthy eating. In addition, a mother/daughter sex communication booklet was included in the educational program. The following section describes the actual iterative adaption procedure for these modifications.

Preconception Counseling Program for Reducing GDM Risk Video Creation.

The project contracted with an American Indian woman-owned video production company with over 20 years of experience in AI/AN health communications. Partnerships developed with an urban Indian health organization in a Great Plains state and production access to a tribe in the Southeast, provided unique opportunities to recruit AI/AN mothers and daughters and AI/AN women with a history of GDM for on-screen talent. Access to “real talent” versus hired actors, contributed to the authenticity of the production experience and final product. The video script was first drafted by the AI/AN-owned production company and then underwent several rounds of iterative editing by the project team for content and production company experts for “acceptability” and “likability”. For example, on-screen text was added in several portions to reiterate important messages and the first several minutes of the video highlight actual stories from AI/AN mothers and daughters to connect viewers more intimately with the messages and reclaim the oral story-telling tradition of tribal communities as recommended by our key stakeholders.

*“I just think it’s awesome, because I know when we get something that’s made for us and that looks like us, and has tribal stuff like us, then it’s not only very attractive to us, but it feels like it’s for us. It feels like it’s exclusive to us, and I’d rather read this than let any other blonde chick read it, because this has tribal stuff on it and it’s not for you.”* (Mother of girl at-risk for GDM)

Preconception Counseling Program for Reducing GDM eBooklet Creation.

**Fig. 1:**
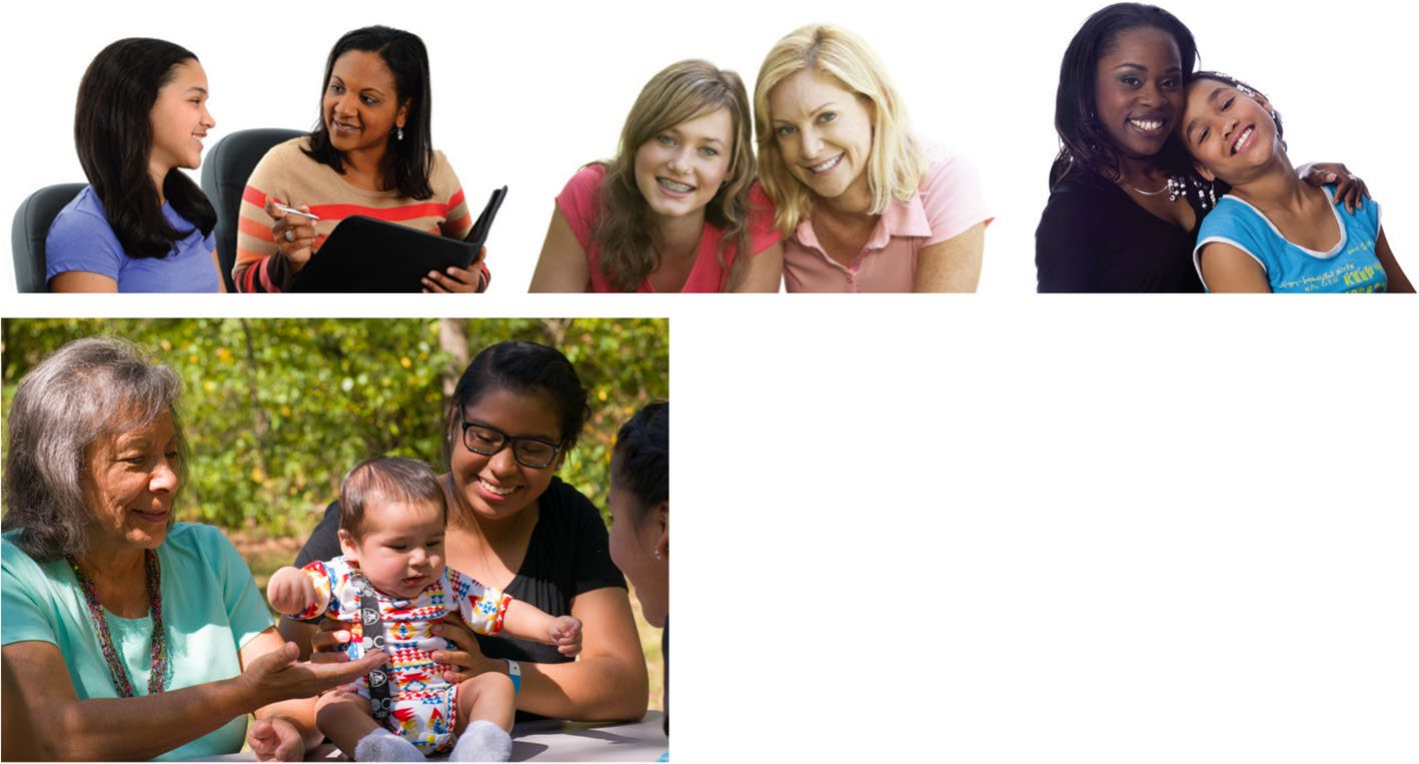
Images of mothers and daughters adapted to include grandmother for intergenerational focus as recommended by tribal voices for Stopping GDM

**Fig. 2:**
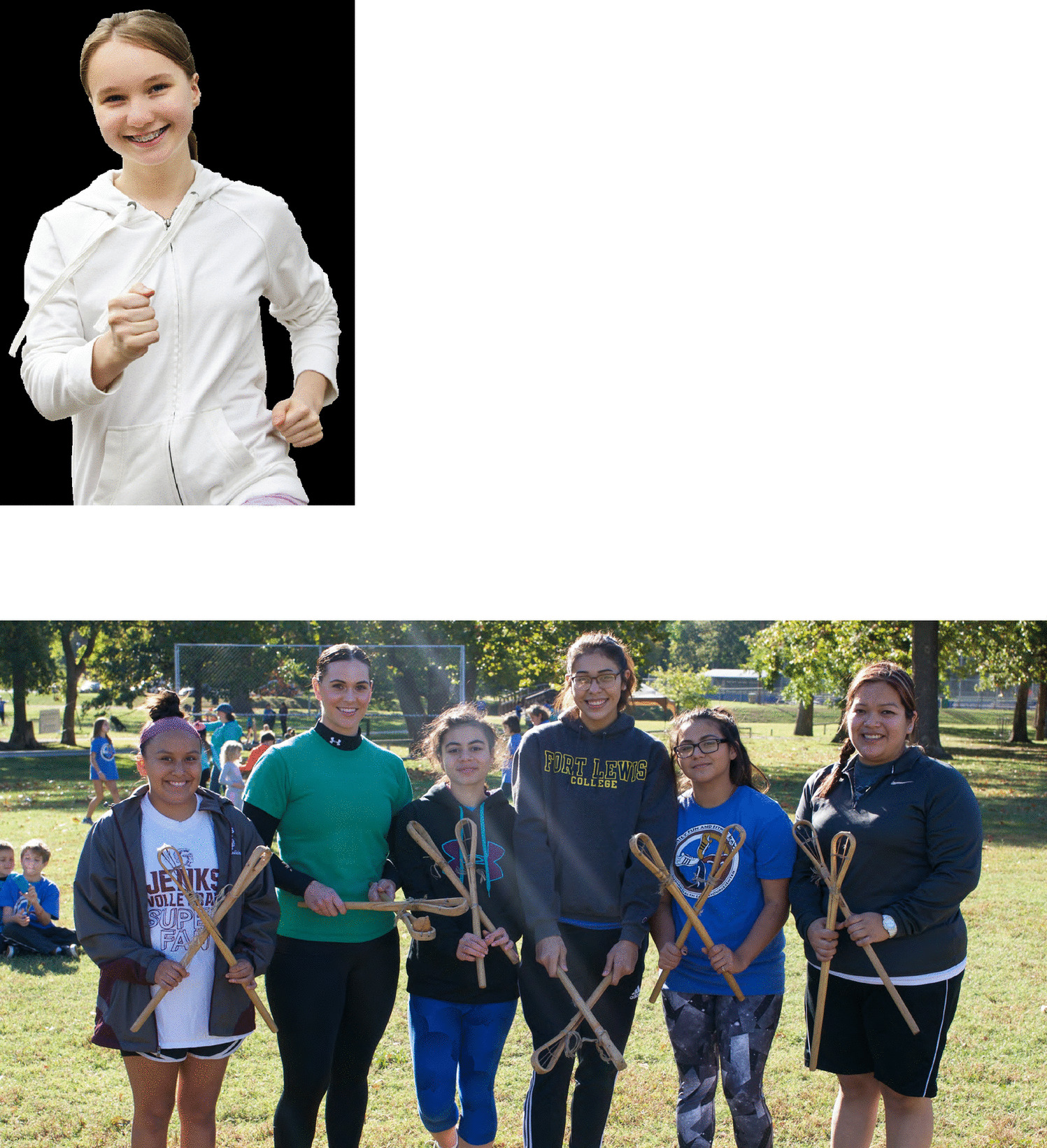
Image depicting physical activity adapted to include traditional game group physical activity as recommended by tribal voices for Stopping GDM

To create an eBooklet that was relevant to diverse tribal audiences, the graphic design team sought to include imagery representative of and appealing to diverse AI/AN communities. For example, we used traditional colors and symbols (e.g., the four directions) and images of multiple generations of women in a family, as exemplified in Fig. 1. As another example, we changed images related to physical activity to be culturally relevant for AI/AN communities. In Fig. 2, we show an AI/AN girls team of a traditional game called stickball, a forerunner of lacrosse.

In designing the eBooklet, the team also considered the needs of low literacy populations and a recommendation to use less directive language voiced by our stakeholders. To address this, design choices of increased white space, supporting graphics, and photographs were used to make the overall product more consumer-friendly, and less academic in nature. Appropriate supportive language was incorporated, rather than merely directing our AI/AN audience what to do. For example, in the eBooklet, we added “You have a right to…” at the beginning of the following sentence, “… make sure you have a healthy pregnancy for yourself and your baby.” In addition, a companion sex communication booklet for the mothers was included in the online program to help address our finding of lack of communication on reproductive health between mothers and daughters.

### Thematic discussion

This manuscript presents the overlapping themes from the many voices of the diverse stakeholder groups that informed the adaptation of *Stopping GDM*. The salient components of the four major converging themes that were addressed included the following: limitations in awareness and knowledge of GDM among our target audience of AI/AN adolescents and their mothers as well as a lack of AI/AN tailored GDM resources; acknowledging traditional AI/AN values and the vast diversity of traditions and culture among AI/AN tribes; promoting skill-building for healthy lifestyle choices related to diet and physical activity while empowering girls to make safe and healthy choices regarding their reproductive health; and the lack of communication about reproductive health between AI/AN mothers and daughters and between AI/AN women and health care professionals.

Most studies related to GDM awareness and knowledge have been quantitative and conducted with pregnant women. Studies among pregnant women in India, a multi-ethnic cohort in Australia, Turkey and Samoa [43–46] have demonstrated a low level of awareness and knowledge regarding GDM with the authors concluding that more education is needed to increase awareness [47]. In contrast, research conducted among Polish women of child-bearing age [48] and pregnant women [47] has shown a moderate and even high level of knowledge of GDM and its risk factors, respectively. Nonetheless, Lis-Kuberka and Orczyk-Pawiłowicz highlighted the need to educate women *prior to pregnancy* on the risks associated with the development of GDM and the increased post-partum risk for the development of T2D [48]. Thomas pointed to the risks to the progeny of pregnancies affected by GDM that include the development of T2D and obesity and how education programs could reduce risk not only for the mother but also for the baby [47, 49]. *Stopping GDM* has been designed to address these very gaps. Furthermore, an ethnographic study among First Nations women in Canada who had pre-existing T2D prior to pregnancy or GDM also demonstrated a lack of awareness and knowledge of GDM. Notably, the lack of Indigenous-specific resources related to GDM was another important finding of this Canadian qualitative study, bolstering the similar finding from the *Stopping GDM* stakeholders [50]. Raising awareness of GDM and risk factors among Indigenous women is a promising first step in a comprehensive culturally responsive and tailored preconception counseling education program [45].

Culturally appropriate diabetes education programs have been shown to be effective within other disproportionately affected populations [51]. Culturally tailoring education materials and programming for an AI/AN audience is known to have a greater impact on its acceptance and engagement when compared with materials and programming that have not been tailored for this audience [52]. As in other evidence-based AI/AN-specific diabetes prevention programs, these findings support inclusion of community and traditional AI/AN values and images [53]. Moreover, the ADCES endorses the delivery of culturally relevant resources and services and collaboration with communities to build culturally appropriate interventions and states “education materials and programs must require thoughtful consideration of many factors, including cultural characteristics, norms, experiences, values, behavioral patterns, beliefs, values, as well as historical, environmental and social forces” [13].

Providing culturally tailored information is imperative. Therefore, we chose a broad composite stakeholder approach of expert, professional and lay panels in our focus groups and interviews for qualitative data collection. Voices from key stakeholders early in program adaptation were critical to assure that our *Stopping GDM* program was developed with the voices and perspectives of the priority audience represented [30]. These themes have been incorporated into our *Stopping GDM* program to address an important gap in an area of health research and education programming that is virtually non-existent, but greatly needed. Indeed, drawing on traditional knowledge, wisdom and values honoring the roles of women, family and the community in tribal society, as well as other traditional cultural practices, is cited by our key stakeholders as an essential and critical element for reproductive health education for AI/AN populations.

The same behavioral change model, the Expanded Health Belief Model (EHBM), used for *READY-Girls*, served as the underlying theoretical framework for *Stopping GDM*. This model is relevant, because understanding a cultural group’s health beliefs, health behaviors, or barriers affects their responses to healthcare [54]. Two key constructs of this model include perceived severity and perceived susceptibility – both of which make up the “perceived risk” [26]. Our findings suggest “perceived risk” is low in the AI/AN audience, likely due to the lack of comprehensive awareness and education campaigns focused on GDM, especially prior to pregnancy. The EHBM construct “self-efficacy” is further support for empowering girls to make healthy choices. Expansion of this construct of “self-efficacy” beyond girls as individuals to the whole community is critical. Providing a support network which includes the girls’ mothers, significant female caregivers, and the community also aligns with traditional and cultural norms. Building healthier communities through inclusion of families and other role models will create an environment that supports healthy choices and opportunities for girls (and for all community members) regarding their reproductive health, food, and activity habits. Inclusion of family and community members has also been identified as an important approach to establishing cultural competency when conducting health research in AI/AN communities [54]. Furthermore, reconnecting girls with their mothers and women to their communities has not only informed the *Stopping GDM* program but may also promote community and cultural resiliency.

Strengths of our process in the development of *Stopping GDM* include the range of our stakeholders, representative of the diversity of AI/AN tribal communities and our target audience – rural and urban, young and old, professional and lay, mothers and daughters – from across the US. In addition, we included health professionals, elected tribal officials, and AI/AN health program administrators with expertise in the delivery of health care and health promotion education to tribal members, as well as experts in key content areas including GDM, pediatric diabetes, adolescent health and reproductive health. However, the combination of unforeseen delays in the transcription and qualitative analysis of the focus groups, the need for scientific content adaptation from an emphasis on metabolic control of type 1 diabetes (T1D) and T2D prior to pregnancy to primary prevention of GDM, and an aggressive timeline in order to complete the full adaptation of *Stopping GDM* for the RCT allowed limited time for reflection and incorporation of all the feedback from our stakeholders.

## Conclusion

Given that these themes are reflective of multiple stakeholder groups, they are salient and robust and provide the content validity for the program. Perspectives from these key stakeholders have been used to guide the development of a culturally responsive GDM risk reduction program for AI/AN girls. Prior to the multi-site RCT, beta testing was conducted among AI/AN mother–daughter dyads recruited from an urban Indian health program to understand the efficacy and ease of using the online portal delivery system of the *Stopping GDM* intervention [55]. The intervention has also been tested in a multi-site RCT to evaluate the *Stopping GDM* program with AI/AN girls at-risk for GDM and their mothers and is now being disseminated widely. The *Stopping GDM* materials are available online at www.stoppinggdm.com.

## Data Availability

The datasets used and/or analyzed during the current study are available from the corresponding author on reasonable request.

## Abbreviations

ADCES: Association of Diabetes Care and Education Specialists
AI/AN: American Indian/Alaska Native
COMIRB: Colorado Multiple Institutional Review Board
EHBM: Expanded Health Belief Model
GDM: Gestational diabetes mellitus
PC: Preconception counseling
T1D: Type 1 diabetes
T2D: Type 2 diabetes
RCT: Randomized controlled trial
